# Recent developments in StemBase: a tool to study gene expression in human and murine stem cells

**DOI:** 10.1186/1756-0500-2-39

**Published:** 2009-03-10

**Authors:** Reatha Sandie, Gareth A Palidwor, Matthew R Huska, Christopher J Porter, Paul M Krzyzanowski, Enrique M Muro, Carolina Perez-Iratxeta, Miguel A Andrade-Navarro

**Affiliations:** 1 Ottawa Health Research Institute, 501 Smyth Road, Ottawa, ON K1H 8L6, Canada; 2 Max-Delbrück Center for Molecular Medicine, Robert Rössle Str. 10, 13125 Berlin, Germany

## Abstract

**Background:**

Currently one of the largest online repositories for human and mouse stem cell gene expression data, StemBase was first designed as a simple web-interface to DNA microarray data generated by the Canadian Stem Cell Network to facilitate the discovery of gene functions relevant to stem cell control and differentiation.

**Findings:**

Since its creation, StemBase has grown in both size and scope into a system with analysis tools that examine either the whole database at once, or slices of data, based on tissue type, cell type or gene of interest. As of September 1, 2008, StemBase contains gene expression data (microarray and Serial Analysis of Gene Expression) from 210 stem cell samples in 60 different experiments.

**Conclusion:**

StemBase can be used to study gene expression in human and murine stem cells and is available at .

## Findings

Stem cells are unique in that they are able to differentiate into any number of different cell lineages. The ability to reprogram undifferentiated stem cells into a specific cell type is currently a source of intense study as their potential therapeutic applications are many [1]. However, the mechanisms of stem cell differentiation remain largely unexplained [2].

To facilitate the discovery of genes with functions important to the control of stem cell fate, a collection of gene expression measurements in samples of stem cells and derivatives from mouse and human (mostly Affymetrix microarray data) was produced. These data were generated within the framework of the Stem Cell Genomics Project and funded by the Canadian Stem Cell Network. The StemBase database was created as a public repository of these data (; [3]). StemBase has evolved from a simple search interface to a more complex analysis tool [4]. Here we briefly introduce the database and describe in detail the querying features recently added (namely, complementary analysis tools to study gene co-expression and view the data in genomic context), which have expanded considerably the functionality of the database.

Other recently developed repositories of stem cell gene expression data have a narrower scope than StemBase, focusing on human embryonic stem cells [5] and murine blood stem cells [6]. In contrast, StemBase has a wider scope as it includes data from mouse and human cells, and collects data from as many types of stem cells and their derivatives as possible.

## Data sets and formats

The gene expression data in StemBase are arranged in a hierarchy of three levels: experiment, sample and replicate. Every experiment has a series of samples, usually comprising a unique set of experimental conditions and most samples have three biological replicates. Experiments compare either gene expression of particular stem cells under different conditions, stem cell enriched tissues, or stem cells to their differentiated derivatives. Some detailed experiments consist of 7- or 11-point time series that follow stem cell differentiation.

The majority of gene expression data are derived from Affymetrix GeneChip(R) DNA microarrays (Table 1; the complete up to date list is online at ). As of September 2008, there are 50 human samples (analyzed on the HG-U133 chip series), 151 mouse samples (analyzed on either the MOE430 or MG-U74v2 chip series) and three rat samples (analyzed on the RAE230 chip series). Affymetrix CEL files were normalized using the MAS5 algorithm.

**Table 1:** Samples in StemBase by tissue, species and platform.

	Affymetrix		SAGE		
		
	mouse	human	rat	mouse	total
	
adipose	1				1
blastocyst	4				4
calvaria			3		3
cancer	4	3			7
chondroblast	2				2
embryonic	47	2		2	51
epithelial	1				1
fibroblast	7				7
hematopoietic	4	18			22
kidney		1			1
mammary	10				10
mast cells	2				2
mesenchymal	14				14
muscle	34	15		2	51
neuronal	12	9		2	23
osteoblast	7				7
retinal	2	2			4
total	151	50	3	6	210

In addition to microarray experiments, StemBase contains data from six SAGE libraries, which correspond to differentiated and undifferentiated stages of three murine stem cell lineages.

StemBase is implemented in a LAMP environment (Linux, Apache, MySQL, PHP). It runs on an Ubuntu 8.04 Server using Apache 2 with the code written in PHP 5.2.4 and using MySQL 5.05 as a database.

## Use of StemBase

Samples and experiments can be accessed individually or as a group selected by species, tissue types, cell ontology terms or cell lines. All data files (raw and processed data) are publicly available for download from either StemBase itself or through the Gene Expression Omnibus (GEO) database at the NCBI [7].

StemBase also provides tools for basic analysis of the microarray data. As most of the tools require either a probe set and/or sample identifier to begin with, simple widgets have been incorporated into the sidebar to allow a user to determine which probe set/chip platforms are associated with a specific gene or the list of identifiers of samples from specific tissues.

To provide access to these functions StemBase has a menu with three items.

1. "Browse" gives access to a list of all the experiments in the database.

2. "Search" gives access to three options for retrieving sets of samples or probes: "Simple" search allows selecting samples or experiments by words contained in their descriptions, "Advanced" search permits using one or more terms for the selection of samples or experiments, and "Find a Probe" finds probe sets according to associated gene identifiers (for advanced searches we recommend using the NetAffx web site from Affymetrix [8]).

3. "Analysis" gives access to tools to retrieve and display gene expression data. These are described in the following paragraphs.

### Exploring probe set expression across samples: Sample vs. Gene

The Sample vs. Gene analysis tool allows the user to obtain the expression levels of a series of probesets and SAGE tags in selected samples. Samples can be selected by defining fields such as species, chip, cell type, or experiments. Probe sets and SAGE tags can be selected by defining fields such as probe set identifiers, SAGE tag sequences, gene symbols or Gene Ontology (GO) identifiers.

**Figure 1:**
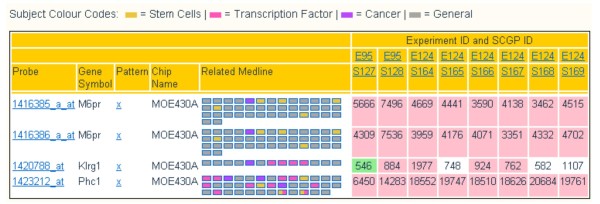
**Sample vs Gene output**. The query used was: species "Mouse", Experiment identifiers "E95 E124", Gene symbols "phc1 m6pr klrg1" (three murine genes that are encoded in a region of chromosome 6). The two experiments selected comprise a total of eight samples, run in the Affymetrix MOE430A chip. The three genes are represented by four probe sets (two for gene M6pr). The numbers indicate the hybridization values reported for each probe set in each sample with background colour indicating MAS5 calls (pink for present, green for absent, white for marginal). The "Related Medline" column contains links to Medline abstracts related to each gene, coloured according to the legend at the top of the graphic: transcription factor functionality is indicated for Klrg1 and Phc1, as well as a relation to stem cells for M6pr and Phc1.

**Figure 2:**
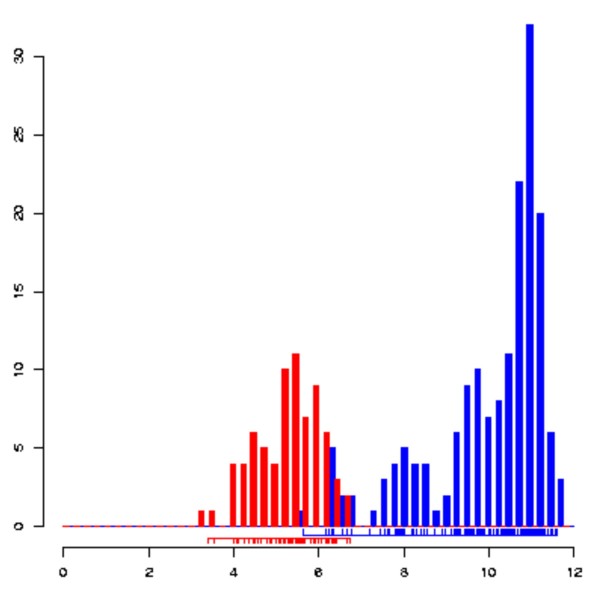
**Pattern of expression**. If a probe set behaves as a marker for a subset of samples in StemBase (that is, its level of hybridization is distinctively higher in that subset of samples than in the rest) its link in the "Pattern" column in Figure 1 will lead to details on this marker-pattern. These include a histogram (as displayed) indicating the number of samples versus hybridization for the samples with high and low expression of the gene, respectively. In this case, the probe set for gene Phc1 (1423212_at) exhibits high expression in murine embryonic samples, P19 embryonic carcinoma and osteoblasts (blue), and low expression in hematopoietic, bone marrow, skin, muscle, mammary and retinal stem cells and derivatives (red).

The results provided by Sample vs. Gene are lists of probe sets and SAGE tags (Figure 1). Each probe set is linked to the NetAffx [8] information page, annotated with the gene symbol associated with the probe set, and, in the case of probe sets in the MOE430 series, linked to the Marker Server: a compilation of genes and expression patterns of putative markers generated for a selection of the data in StemBase [9] (Figure 2). Probe sets can be further annotated with related PubMed articles deemed relevant [10] and coloured by their content, as well as with GO annotations from NetAffx or derived by data mining [11].

### Finding relations between probe sets: correlation and mutual information

An important application of gene expression studies is finding functional relationships between genes from their related patterns of expression [12]. StemBase facilitates this analysis by providing two measurements of expression relatedness between probe sets across a selected set of samples: correlation and mutual information. Both measurements evaluate the similarity of expression of two probe sets, which implies co-expression of their corresponding genes providing evidence that they share common functions.

**Figure 3:**
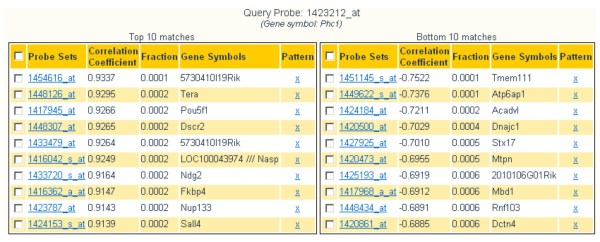
**Correlated genes**. We examined the probe sets with the highest Pearson's correlation with the probe set associated to gene Phc1 in MOE430A, 1423212_at, described in Figures 1 and 2. The tables report the 10 probe sets with highest positive (left) and negative (right) correlation. Among the positively correlated genes we can find Pou5f1/Oct4, a well known embryonic stem cell marker.

The correlation function computes either Pearson's or Spearman's correlation coefficients. In both cases, the returned values rank between +1 and -1 indicating positive and negative correlation, respectively. Positive correlation indicates co-expression, but negative correlation is also informative as exclusive expression of two genes may indicate that, for example, one gene is suppressing the other. The expression values for a query probe set are compared to all other probe sets in the chosen microarray platform across all samples in the database. Results are returned in two ranked lists for positively and negatively correlated probe sets (Figure 3).

Mutual information is used to measure the mutual dependence between the expression profiles of two probe sets. It is calculated from MAS5 expression calls (Present/Marginal/Absent) of a user's query probe set and all other probe sets on the same platform. The tool returns positive values normalized to 1, where values close to 1 indicate similarity of gene expression.

These three measurements are complementary and therefore all are indicated for use in an exploratory analysis. For example, each calculation identifies a different probe set most correlated with probe set 1416967_at (transcription factor Sox2) in all mouse samples hybridized to the MOE430A array: 1449374_at (Pipox) with Pearson coefficient 0.8390, 1421883_at (Elavl2) with Spearman coefficient 0.8891, and 1423424_at (Zic3) with a Mutual Information score of 0.7139 (normalized).

### Visualizing expression data on genomic regions: Genome Viewer

The Genome Viewer was designed to graphically represent the mapping of the SAGE tags to genomic positions. This allows comparing the results from SAGE libraries with microarray data and other genomic features such as genes and EST data in particular genomic positions.

We use the UCSC Genome Browser [13] to represent the location and expression levels of SAGE tags and probe sets. StemBase provides the option of choosing a specific platform, either microarray chip or SAGE data, a sample and a chromosome. The query can be further narrowed to particular positions on the chromosome. Then, a custom link to the UCSC Genome Browser is generated, which displays the queried data.

**Figure 4:**
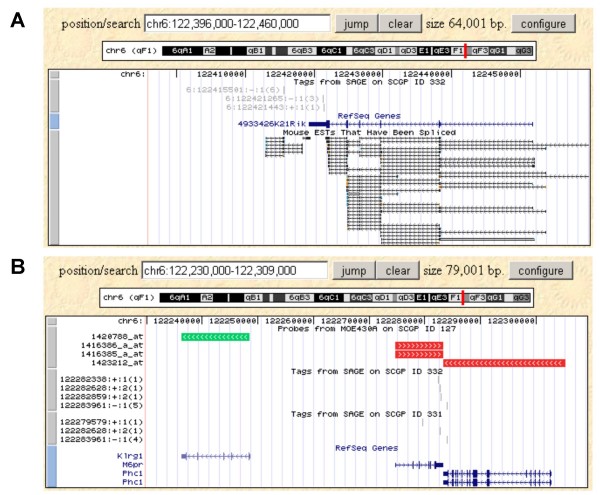
**Genomic viewer**. **(A)** Genomic view of SAGE data. Sample S332 is a SAGE library obtained from differentiated neural stem cells. The view using the UCSC Genome Browser displays three SAGE tags (grey) whose identifiers indicate their chromosome and start position (in the Mouse Genome assembly from February 2006, version mm8) followed by sense, number of mapping locations, and tag counts in the library. The RefSeq Genes track shows only one gene (of uncharacterized function) in the region. Two SAGE tags overlap with the 3'UTR of the gene suggesting the expression of this gene and of an anti-sense transcript to this gene, respectively. The leftmost tag suggests the expression of a second transcript downstream this gene consistent with the data shown in the spliced ESTs track. **(B)** Combined view of SAGE and microarray data. The view shows another region of murine chromosome 6, which encodes the three genes analyzed in Figure 1, indicated by the RefSeq genes track (blue): Klrg1, M6pr and Phc1. In addition to the data from the SAGE library displayed in part A of the figure, additional tracks show data from another two samples. Those are its counterpart SAGE library (S331, neural stem cells) and a sample of DNA microarray of embryonic stem cells undergoing differentiation (S127). All three samples suggest lack of expression of the Klrg1 gene in these tissues: no SAGE tags overlapping with it and probe set 1420788_at for a transcript of this gene indicates no gene expression (green). The other two genes are expressed in the conditions represented.

SAGE tag sequence positions are mapped using the current murine genome version [14]. Since tag sequences are only 21 nucleotides long, some are mapped to multiple genomic locations. The Genome viewer will display only SAGE tags mapped to at most four locations. The tags are visualized as a separate track in the UCSC Genome Browser with a label indicating each tag's position, direction, number of mapping positions and counts in the given library (Figure 4A). The sequence of any tag can be retrieved by clicking on its label.

Microarrray probe set positions are derived from the UCSC mouse genome annotation data. Only probe sets that can be reliably located on the genome are shown. Probe sets are visualized as a track in the UCSC Genome Browser and colour-coded by their MAS5 call values (Present – red, Absent – green, Marginal – yellow, Undetermined – grey). A combination of microarray data and SAGE library data can be displayed in the same view (Figure 4B).

## Conclusion

StemBase is a large resource of DNA microarray gene expression data generated from stem cell related samples. As such, it contains information relevant to the study of stem cell function and differentiation in a variety of human and mouse tissues.

While DNA microarrays are an effective tool for large-scale gene expression experiments, the large quantity of data generated makes effective analysis problematic. In recent years, several software packages have been developed to assist the analysis of microarray results [15], but the process is still time consuming and difficult for those not altogether familiar with microarrays or the software packages in question. For this reason, we implemented web-based tools in StemBase that allow researchers to easily analyze these domain-specific data without requiring any additional software. StemBase's tools facilitate the exploration and visualization of the expression of particular genes across samples of stem cells and derivatives, finding genes with particular patterns of expression across those samples, and linking the results to information from external databases. The addition of further samples to expand the current database (both locally generated and from worldwide resources) is a continuing goal, as is providing support for further analysis of current gene expression data.

## Availability and requirements

**Project name**: StemBase

**Project home page**: 

**Operating system(s)**: Platform independent

**Programming language**: PHP

**Other requirements**: StemBase is optimally viewed with the Mozilla Firefox web browser.

**License**: none

**Any restrictions to use by non-academics**: none

Several tutorials which include different aspects of the use of StemBase are available within the Stem Cell Network Microarray Analysis Course .

## Competing interests

The authors declare that they have no competing interests.

## Authors' contributions

RS and GP participated in the programming of the database and in drafting the manuscript. MH, CP, PK, EM, and CPI designed and helped to implement parts of the database. CPI and MA participated in drafting the manuscript. MA coordinated the development of the database. All authors participated in the design of the database, and read and approved the final manuscript.
